# The Usual Suspects 2019: of Chips, Droplets, Synthesis, and Artificial Cells

**DOI:** 10.3390/mi10050285

**Published:** 2019-04-27

**Authors:** Christoph Eilenberger, Sarah Spitz, Barbara Eva Maria Bachmann, Eva Kathrin Ehmoser, Peter Ertl, Mario Rothbauer

**Affiliations:** 1 Institute of Applied Synthetic Chemistry, Institute of Chemical Technologies and Analytics, Faculty of Technical Chemistry, Vienna University of Technology, A-1060 Vienna, Austria; christoph.eilenberger@tuwien.ac.at (C.E.); sarah.spitz@tuwien.ac.at (S.S.); 2 Austrian Cluster for Tissue Regeneration and Ludwig Boltzmann Institute for Experimental and Clinical Traumatology, Allgemeine Unfallversicherungsanstalt (AUVA) Research Centre, A-1200 Vienna, Austria; barbara.bachmann@tuwien.ac.at; 3 Institute of Synthetic Bioarchitectures, Department of Nanobiotechnology, University of Natural Resources and Life Sciences Vienna, A-1190 Vienna, Austria

**Keywords:** synthetic biology, microfluidics, high throughput, artificial cells, protein synthesis, cell-free synthesis, droplets, bottom-up, top-down

## Abstract

Synthetic biology aims to understand fundamental biological processes in more detail than possible for actual living cells. Synthetic biology can combat decomposition and build-up of artificial experimental models under precisely controlled and defined environmental and biochemical conditions. Microfluidic systems can provide the tools to improve and refine existing synthetic systems because they allow control and manipulation of liquids on a micro- and nanoscale. In addition, chip-based approaches are predisposed for synthetic biology applications since they present an opportune technological toolkit capable of fully automated high throughput and content screening under low reagent consumption. This review critically highlights the latest updates in microfluidic cell-free and cell-based protein synthesis as well as the progress on chip-based artificial cells. Even though progress is slow for microfluidic synthetic biology, microfluidic systems are valuable tools for synthetic biology and may one day help to give answers to long asked questions of fundamental cell biology and life itself.

## 1. Introduction

Investigation on function, structure, and dynamics of cells at the molecular level will be the key to understand fundamental uncertainties regarding the definition and the origin of life. Synthetic biology is an emerging discipline that attempts to synthesize, re-engineer, and manipulate biological systems under very controlled conditions to better understand nature [1]. This interdisciplinary field aims to design and synthesize unnatural (bio)chemical structures using bottom-up or top-down approaches, on a genomic, proteomic, or cellular level, and to re-composite and manipulate consisting biological systems [2]. Essentially, synthetic biology is based on well-characterized and functional DNA building blocks, which assemble into newly designed biosystems [3]. From the engineering point of view, classical engineering development cycles can be used to facilitate synthetic biology processes based on four steps that comprise the design, the construction, the testing, and the analysis of an artificial system in relation to the functional and structural properties of the natural system [4,5,6]. These key steps, also referred to as design–build–test–learn cycles, include techniques such as DNA synthesis, DNA assembly, DNA transformation, cell culture, and phenotypic analysis, which often require costly and labor-intensive manual processes, consume large amounts of expensive reagents such as enzymes and synthetic DNA, are limited in throughput, and have poor reproducibility even though performed under controlled experimental conditions [7,8].

Microfluidic systems overcome many of the drawbacks; they can even improve and refine existing systems because they allow control and manipulation of liquids on a microscopic scale, and they are capable of high throughput, low reagent consumption, and automation. Commercial microfluidic devices are becoming increasingly commonplace in the lab, with devices popular in the handling of DNA and RNA such as fragment analyzers used in next-generation sequencing workflows [9].

In this context, microfluidic and microfabrication technology has the potential to provide the next generation of analysis tools capable of inexpensively testing a large number of newly designed biomolecules, proteins, and artificial cells [10]. With fabrication techniques that originated from the microelectronics industry, photo- and soft-lithography have become a dominant trend in recent years because of its fast and inexpensive fabrication of microdevices using polydimethylsiloxane (PDMS), thermoset composites, and thermoplastics [11]. But it has to be considered that the fabrication method is mostly determined by the existing infrastructure, fabrication speed, desired resolution, and fabrication material [12,13]. The majority of microfluidic devices used for synthetic biology can be categorized into channel-based microfluidic and droplet-based microfluidic designs [14]. Channel-based microfluidics can provide a well-defined and long-term environment for real-time observation, where slight changes of cell behavior can be captured for the purpose of quantitative analysis [15]. On the other hand, droplet-based microfluidics can generate complex sample droplets at an extremely high throughput with controllable size and core-shell properties as a result of phase separation. In the simplest case, a water droplet is protected by a surrounding oil phase; they can be used as isolated bioreactors for living cells or even for scale-down of biochemical reactions [16]. Microfluidic technologies and lab-on-a-chip devices have been developed independently, but their transfer into the field of synthetic biology will help inspire and kickstart completely new assembly and assessment strategies for synthetic bioarchitectures [17]. 

In this review we aim to critically reflect on the latest progress and advances of microfluidic synthetic biology over the last year (2018–2019) including design and development of microfluidics and its applications to build and understand synthetic biosystems. In particular, devices supporting cell-based and cell-free protein expression are highlighted because these systems outweigh more exotic, and therefore rarer chip-based, approaches.

## 2. Cell-Based Protein Synthesis—A Ménage à Trois of Droplets, Digital Microfluidics, and Cells

Synthetic biology approaches provide an important tool for on-demand control of gene expression mechanisms in cellular organisms. Applications allowing the user to engineer cellular pathways are not only vital for optimizing biotechnological processes but also for understanding physiologic and pathologic mechanisms in cell biology. The addition of microfluidic devices to the equation enables the user to (i) guide cellular microenvironments using automated feedback algorithms, (ii) entrap cells in droplets to sort for the most valuable strains, and (iii) conduct sophisticated experiments, for instance, to fine-tune inducer concentrations or to observe prokaryotic and eukaryotic cells in a dynamically changing microenvironment [18].

**Figure 1:**
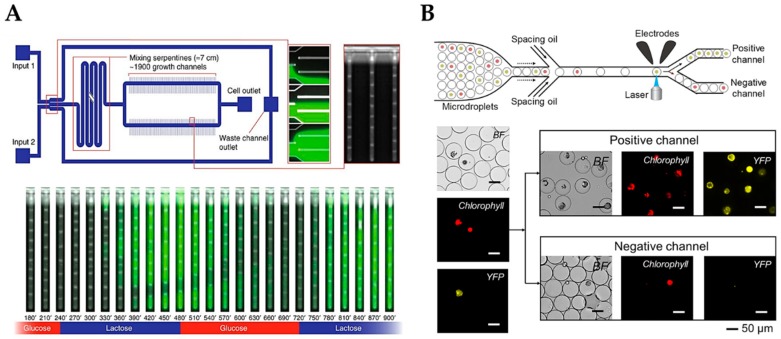
(**A**) Chip for single-cell analysis of gene regulation under dynamically controllable conditions with the integrated software algorithm. (Reproduced from [19] with permission from Nature Publishing Group). (**B**) Droplet-based microfluidic analysis system that can screen single plant cells in a controllable soluble microenvironment. (Reprinted with permission from [21]; Copyright 2019 American Chemical Society).

Dynamic changes of the environment can be generated by fluctuating lactose supply to lac-operon-controlled *Escherichia coli (E. coli)*. To monitor such changes in single cells and their progeny, Kaiser et al. [19] combined a dual-input Mother Machine Chip (DIMM, shown in Figure 1A) with a Mother Machine Software Analyzer (MoMA) algorithm. The application of the DIMM microfluidic chip offers several advantages over common in vitro cultivation, namely (i) employing dynamic changes in substrate type and concentration, (ii) observing the gene regulatory response in each single cell, and (iii) tracking gene expression changes over time. By utilizing the powerful MoMA software, capable of segmenting and tracking cells in phase-contrast images, the researchers identified several fascinating novel features of lac operon induction in *E. coli*. Nonetheless, even though this setup provides a powerful tool for dynamic gene regulation studies, its capabilities were demonstrated with a widely used standard host organism and promoter, therefore presenting a mere proof-of-principle study. Applying this knowledge to test the stochastic properties of synthetically engineered inducible promoters would pose an important next step in the development of such microfluidics for synthetic biology. A good example of such a system applied to mammalian cells was recently published by Postiglione et al. [18], proving how cultivation of synthetically engineered mammalian cells can be combined with control engineering for automated adjustment of inducer concentration and, thus, protein expression. Inserting not just microorganisms but mammalian cells within such a microfluidic setup provides multiple exciting possibilities for cybergenetics to improve both biotechnological production as well as understanding pathways in cellular development and differentiation. Even though the microfluidic device is not new and has been initially developed by Kolnik et al. [20], it enables shear-free cultivation of mammalian cells with automated cell loading and medium exchange. The device is housed in a setup that optically determines the accumulation of expressed fluorescent reporter molecules. Further, it automatically adjusts the expression to a reference level by varying inducer concentration using two syringes by a feedback-loop control. The system was not only successfully applied to chinese hamster ovary cells, the standard mammalian workhorse for producing recombinant proteins, but also in mouse embryonic stem cells. The opportunity to control gene expression in complicated mammalian cells promises enthralling new opportunities for fundamental cell biology as well as thrilling future insight into human medicine.

Alternative to the merits outlined above, microfluidic devices can also be used for active expression screening and cell sorting. In droplet generators individual synthetically engineered cells are encapsulated in droplets by utilizing the laminar flow properties of fluid handling at the microscale. Laminar flows of an immiscible fluid perpendicular to the droplet-generating channel lead to the formation of hydrophilic droplets within a hydrophobic carrier fluid. By addition of cells to the hydrophilic fluid, single cells can be encapsulated in these droplets and later on sorted by specific properties such as fluorescent gene expression. Microfabricated fluorescence-activated cell sorter (µFACS) systems with inline droplet generators offer similar advantages as FACS; however, they eliminate the need for expensive equipment and minimize the probability of channel clogging. One recent example of employing droplet microfluidics for cell sorting has been shown by Yu et al. for plant protoplasts, as shown in Figure 1B [21]. Protoplast fluorescence was detected on-chip by coupling a laser-based optical detection setup with electrodes generating a dielectric force dependent on fluorescent readout. If a droplet containing a protoplast positive for either chlorophyll or yellow fluorescent protein (YFP) passes the optical detection unit, the droplet is steered into the positive channel by activating the electrodes whereas negative droplets are excluded by fluid resistance. The microdevice shows high success rates, as all microdroplets collected in the positive channel contain YFP-expressing protoplasts, and the negative channel mainly features empty droplets or droplets containing ruptured or wild type protoplasts. By adding this microfluidic sorting unit to the experimental palette, possibilities for synthetic engineering of plants are significantly enhanced. Identifying successfully transfected protoplasts prior to strenuous tissue culture undoubtedly can decrease time and cost of experiments, which is quite usable for today’s scientists for obvious reasons. As cheap and nice as these systems may be, droplet generators are often limited to hydrophilic proteins since hydrophobic expression products (e.g., oils for biofuel production) are soluble in the carrier oil. To overcome this downside, Siltanen et al. [22] engineered a platform enabling microfluidic cell sorting and subsequent printing of droplets onto a microwell array. First, droplet-encapsulated yeast colonies were sorted based on similar optical density using dielectrophoretic cell sorting as described above. Subsequently, isogenic colonies were printed onto a microarray consisting of dielectrophoretic traps placed below nanoliter-sized wells. After substrate addition, the hydrophobic carrier oil was aspirated and replaced by humidified air to solve the carrier issues. Finally, successful staining of hydrophobic expression product was enabled by encapsulating the yeast colonies in a hydrogel mesh. Nonetheless, the system would greatly benefit from additional microfluidic upgrades to enable cell culture within the same device prior to as well as after sorting, on-line quantitative fluorescent detection, and carrier replacement.

**Figure 2:**
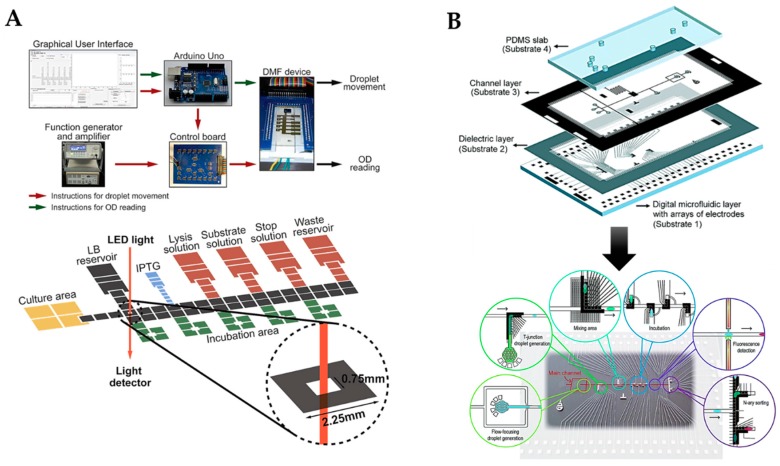
(**A**) Digital microfluidic system with inline analysis module for bacterial cell culture induction and handling. (Reprinted with permission from [24]; Copyright 2019 American Chemical Society). (**B**) An integrated and multilayered droplet-digital microfluidic system for on-demand droplet creation, mixing, incubation, and sorting combining droplet with digital microfluidics. (Reproduced from [25] with permission from The Royal Society of Chemistry).

As an alternative to pressure-driven fluid flow systems, droplets can also be generated and manipulated using digital microfluidics (DMF). In contrast to traditional microfluidics, DMF utilizes alternating currents on an electrode array for moving fluid in the microdevice. Shortly, the liquid is moved on an open-plane device through manipulation of the droplet’s surface tension by electrowetting. For a more detailed description, the reader is referred to an excellent recent review by Jebrail et al. [23]. Digital microfluidics provides several advantages over traditional pump-based systems, as it eliminates the need for bulky lab equipment and allows precise control over the droplet movements including droplet fusion and separation. To demonstrate the applicability of such systems for synthetic biology, Husser et al. [24] recently developed the first automated induction microfluidics system (AIMS; see Figure 2A). This integrative approach offered several advantages, such as (i) automation of bacterial cell culture induction and handling, (ii) reducing the risk of cross-contamination, and (iii) simultaneous screening of multiple cultures. The AIMS featured, amongst others, a cell culture mixing chamber, an absorbance measurement spot, as well as incubation areas for multiple samples. In the experimental setup, a mother droplet with cells was dispensed into the culture area, where it was mixed by alternating vertical and horizontal currents. Upon experimental initiation, the mother droplet was moved to the absorbance measurement spot, and the experiment was automatically initiated if the optical density (OD) exceeded a certain threshold. The AIMS featured two operation modes, (1) automated monitoring of fluorescent protein expression using varied inducer concentrations or (2) screening of expressed enzyme activity using fluorescent reaction products. Even though the freedom of fluid manipulation is undisputable, the biggest downside for the system is that the devices need to still be transferred to a plate reader for fluorescent detection and read-out and, therefore, still lacks some vital parameters for full automation of synthetic biology on a single device. Lastly, droplet and digital microfluidics can be combined by adding a DMF manipulation layer to a classic microfluidic droplet-generating channel microstructure, thus creating an integrated and multi-layered droplet-digital microfluidic (I2DM) system (see Figure 2B). This technology, recently published by Ahmadi et al. [25], relies on pressure-driven microfluidic droplet generation with subsequent digital microfluidic on-demand droplet manipulation. First, single cells are encapsulated in droplets using the pressure-driven droplet part of the device and subsequently merged and mixed with a droplet of inducer fluid on the DMF. Secondly, the droplet containing a single cell is transferred to the incubation region of the device using fluid flow, where it is incubated for 24 h to allow protein expression. Finally, the droplet is then analyzed for cell density using absorbance and sorted through an n-array cell sorting channel. Similar to the previously mentioned devices, this microfluidic setup holds great promise for microfluidic analysis of synthetically engineered cells. However, significant work still needs to be conducted to integrate cell cultivation and allow higher throughput and on-line measurement methods.

Overall, microfluidics proves to be an important tool for synthetic biologists in manipulating cellular systems. The devices offer great advantages such as automated feedback control [18,19], sorting of engineered cells based on protein yield [21,22,25], and on-line detection of cell growth [25,26]. Digital microfluidic devices additionally allow for novel fluid handling operations and process automation. However, despite these many advances and advantages, the technology is still in its infancy. In the future, automated cell cultivation, protein expression, and detection will give rise to an emerging field holding numerous promises.

## 3. Microfluidic Devices for Cell-Free Protein Expression

In recent years, the emergence of cell-free synthetic biology has opened opportunities for studying complex cellular activities in vitro in the absence of heterogenous living cells. This powerful technology allows biological networks to be engineered in a more controllable and less complex experimental setup, which allows rapid prototyping of newly designed gene circuits before implementing them in living cells [27,28]. 

Cell-free protein synthesis (CFPS) systems offer many advantages over cell-based systems, including high protein yield, the generation of soluble and functional proteins without inhibition of regulatory pathways, as well as the possibility of using mRNA fragments directly without any need for cloning [29,30]. Additionally, many proteins are unstable and proteolytically sensitive, which makes a cellular microenvironment a rather harsh environment [31,32]. However, CFPS has been explored for synthetic biology, allowing engineering of biomolecular systems with cell-like behaviors and construction of artificial cell-like structures such as attachment and integration of plasmid-DNA within a hydrogel matrix by chemical manipulation [33,34,35]. 

**Figure 3:**
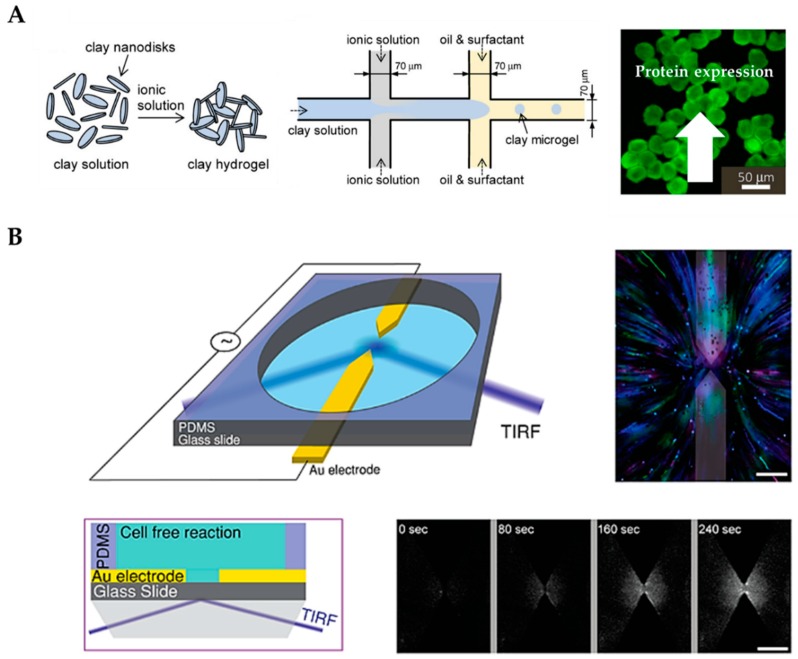
(**A**) Droplet-based clay microgel system with compartmentalized microenvironments capable of high-yield and repeated protein synthesis. (Reprinted with permission from [36]; Copyright 2019 American Chemical Society). (**B**) Droplet-based microfluidic using manipulation of an electric field to manipulate cell-free gene expressions. (Reprinted with permission from [49]; Copyright 2019 American Chemical Society).

Jiao et al. recently developed a clay-based hydrogel system for CFPS by using microfluidic droplet technology to circumvent sophisticated chemical manipulations and to preserve the high protein production of plasmids (see Figure 3A). In this system, electrostatic interactions were involved in both the preparation of the clay hydrogel beads (microgels) and the binding of plasmids to the clay microgels. The microfluidic clay microgel system created compartmentalized microenvironments capable of high-yield and repeated protein syntheses, indicated by a six-fold higher enhanced green fluorescent protein (eGFP) production and a 3.5-fold higher expression rate than traditional solution phase systems [36]. Dynamics of mRNA and protein synthesis are key parameters that are needed to optimize the performance of gene circuits. Thus, real-time monitoring of transcription–translation (TX–TL) dynamics is crucial to acquire information of novel synthesized mRNAs and proteins before implementing them in living cells or in artificial cells. Wang et al. used a microfluidic PDMS device for generating cell-sized single-emulsion droplets by encapsulating a mammalian CFPS reaction and a locked nucleic acid (LNA) probe to investigate the dynamics of mRNA and protein expression. Microfluidic-generated water–oil droplets provide an effective method to reduce sample volume to the picolitre range, compared to the bulk reaction volume of microliters, and they offer the possibility for investigation and characterization of gene circuits in the context of live and artificial cells [37]. 

Nevertheless, an open question in cellular communication is the nature of many cellular cascades and how networks of genes interact to form “oscillations” [38,39,40]. The concentrations of mRNAs and proteins increase and decrease rhythmically with a well-defined temporal period in cells. The oscillations of mRNA and protein concentrations are often caused by transcriptional/translational feedback loops, a mechanism that is referred to as a genetic oscillator [41]. These genetic oscillators can be seen, for example, in cell cycles, circadian rhythms, and inflammatory responses [42]. If the activity of one gene in a feedback loop increases, it activates other genes in the circuit that ultimately inhibit it [43,44]. To extend the lifetime of these transcriptional reactions, microfluidic platforms are ideal, since TX–TL components can be replenished, creating an open system wherein the transcription and translation rates are sustained in a steady-state. Yelleswarapu et al. were able to characterize a two-component oscillator with an activator–repressor motif that utilized native transcription machinery of *E. coli*. The behavior of two individual oscillators as well as the behavior of a coupled network were experimentally investigated on an *E. coli*-based TX–TL system operating under steady-state conditions in a pneumatically actuated bi-layer microfluidic device [45]. Since cell-free protein approaches are not restricted by physical barriers, biochemical reactions can be controlled by external fields such as light [46], magnetic fields [47], and electrochemical transduction [48]. In principle, electric field (E-field) manipulation could be a more rapid and specific method and can be combined with microelectronics. To study these effects in more detail, Efrat et al. designed a PDMS device equipped with gold electrodes for trapping ribosomes, RNA polymerases, nascent RNA, and proteins in an electric-field (see Figure 3B) to induce protein synthesis oscillations by on/off switching of the electric field. The combination of an E-field with compartmentalized cell-free expression created a simple, non-invasive approach for controlling synthetic biological systems with a bioelectronic interface [49]. Apart from that, pulsed electric fields can also utilize deformation of the interface between an aqueous and an oil phase. This demonstrates that droplets containing a cell-free transcription–translation system executing protein synthesis could be generated by an electric field-driven droplet generator in a timely and programmable manner [50]. 

**Figure 4:**
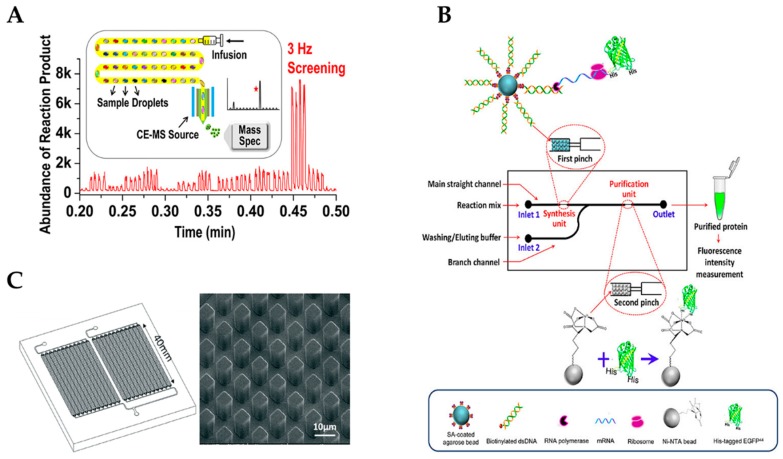
(**A**) Droplet electrospray ionization-mass spectrometry (ESI-MS) methodology for high-throughput biocatalysts screening in Directed Evolution (Reprinted with permission from [53]; Copyright 2019 American Chemical Society). (**B**) A microfluidic chip with integrated cell-free protein synthesis and purification for on-demand production of recombinant proteins. (Reproduced from [58] with permission from AIP Publishing 2018). (**C**) A microfluidic chip with micro-post array for vesicle handling and solid phase cell-free DNA extraction. (Reproduced from [59] with permission from The Royal Society of Chemistry 2018).

Further, the capacity of micro- and nanofabrication in terms of multiplexing and automation combined with CFPS aligns well with the needs of systems biology for high-throughput and fast characterization of cellular functions. Since traditional cell-based protein expression requires multiple days of effort, in contrast, cell-free protein synthesis enhances expression time, as it only requires mixing template DNA with macromolecules and incubation for approximately 2 h [51,52]. The combination of droplet microfluidics interfaced with electrospray ionization-mass spectrometry (ESI-MS) provides an efficient, label-free, high-throughput screening for pharmaceutical biocatalyst applications such as enzyme library screening (see Figure 4A). Especially, industry needs novel analytical methods that are more general, less compound-specific and faster to develop. In a recent paper, throughput was improved to 3 Hz with a wide range of droplet sizes (10–50 nL) demonstrated by using two different transaminase libraries. Droplet-MS showed a significantly faster rate compared to the liquid chromatography–mass spectrometry (LC-MS) method with a 100% match on hit variants, and it showed the capability to perform transcription–translation inside the droplets followed by direct analysis of the reaction mixture by MS. The success of cell-free synthesis in nanoliter droplets suggested great potential for accelerating testing of DNA libraries from 3–4 weeks to 24 h with significant cost savings [53]. Nonetheless, commercial application of microdroplet technology is still rare and is mainly applied to the use of specific equipment in the academic laboratory environment, even though off-the-shelf droplet generators can be purchased from manufacturing companies (e.g., Dolomite, Micronit, Darwin microfluidics, etc.) because of rather high costs (two to three digit € per piece) [54]. Commercial challenges, chip manufacturing, and costs can be read elsewhere [55,56,57]. 

Advances of microfluidics combined with integration of cell-free protein synthesis can also be exploited for therapeutic or diagnostic purposes, for example. Since proteins for point-of-care applications require a certain purity, there remains a need to integrate protein synthesis and protein purification on a microfluidic chip in order to obtain the desired recombinant proteins with a simple operation. Xiao et al. integrated two functional units, a protein synthesis unit and a protein purification unit, into a microfluidic chip for production of a recombinant protein (see Figure 4B) [58]. The first channel was filled with template DNA-modified agarose beads to form a cell-free protein synthesis unit, and the second channel was filled with nickel ion-modified agarose beads (Ni-nitrilotriacetic acid (NTA)) as a protein purification unit. The mixed reaction solution passed through the protein purification unit, where the target protein was captured by Ni-NTA beads. Pure protein was obtained after washing and an elution buffer were introduced to remove non-specific bindings. This device shows the potential to produce single-dose recombinant protein drugs on demand. For the detection of cell-free DNA (cfDNA) in plasma samples of healthy donors and cancer patients, Campos et al. developed a novel microfluidic solid-phase extraction device (µSPE) consisting of a micromachined plastic chip (see Figure 4C) [59]. The chip contained arrays of pillars that were activated with UV/O_3_ to generate surface-confined –COOH functional groups for the selective extraction of cfDNA. One advantage of this chip was the scalability of the target load by tuning the bed size and/or reducing the pillar size to increase the recovery of cfDNA due to reducing diffusion distances. This polymer-based device can be fabricated in a single molding process, negating the need for adding attractional supports and keeping the device and assay costs low for quantification of cfDNA in clinical samples. 

## 4. Microfluidics and Artificial Cells

**Figure 5:**
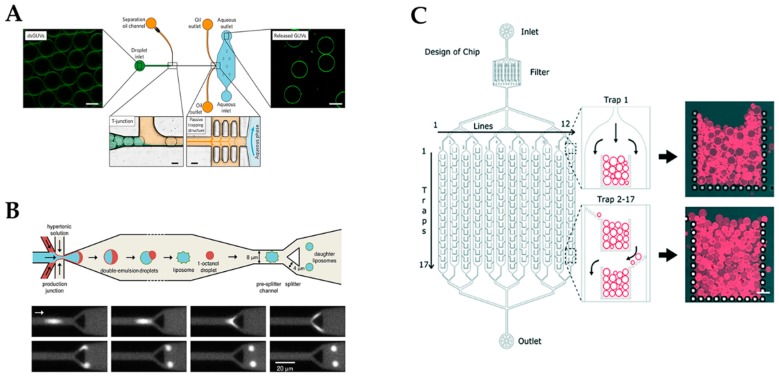
(**A**) A microfluidic device to release the assembled lipid compartments from the surrounding stabilizing polymer droplets into the aqueous phase, showing monodisperse droplet-stabilized GUVs in the oil phase prior to and after release. (Reproduced from [68] with permission from Nature Publishing Group 2018). (**B**) Droplet-based synthetic cell-division simulator that creates two same-site daughter droplets from a single stabilized mother droplet. (Reprinted with permission from [69]; Copyright 2018 American Chemical Society). (**C**) Ultra-high capacity microfluidic device for trapping and vesicle–vesicle interactions of giant vesicles for high-throughput synthetic cell membrane studies (Reproduced from [71] with permission from The Royal Society of Chemistry).

Synthesis of artificial cells disentangled from their complex environments constitutes one of the most important aspects in bottom-up synthetic biology. Bottom-up approaches strive to construct artificial living systems by using non-living matter as initial building blocks. Functionality is achieved by the reconstitution of functional modules from both natural and artificial origins. Through addition of various components, the desired complexity can be built up in a sequential manner, eventually resulting in a truly synthetic living cell [17]. Although living systems feature a high intrinsic complexity, Yewdall et al. [60] recently defined five common hallmarks shared among all of them: compartmentalization, growth and division, information processing, energy transduction, and adaptability. Synthetic biology is trying to address these hallmarks and—with cell-sized compartments representing the most basic unit of a synthetic cell—compartmentalization has become an important topic of investigation over the last years. Especially, cell-sized giant unilamellar vesicles (GUVs) have gained increasing interest because of their natural building blocks as well as their broad applicability as microreactors, biosensors, drug delivery systems, and as artificial cells [61,62,63]. Unfortunately, the need for precise control over critical aspects such as vesicle size, architecture, compartment number, interconnectivity, and functionalization is not met by standard methods such as electroformation and film hydration. With its ability for precision, high-throughput, and controlled fluid handling, as already outlined in the first two sections of this review, microfluidics provide a powerful toolkit for addressing these complex requirements [64,65]. Using droplet microfluidics, Elani et al. [66] were able to generate complex hybrid cellular bionic systems by functionalizing GUVs with functional modules of biological origin. Within the microfluidic device, *E. coli* and several eukaryotic cell lines could be successfully integrated into vesicles. This modification yielded a functional synergy between the encapsulated cell and the vesicle host. While external architecture was able to efficiently shield the cell from its toxic surroundings, the cell acted as an organelle-like module by conferring the artificial cell with its cellular biochemistry. The coupling of cellular and non-cellular pathways was demonstrated by devising a three-step biochemical pathway ultimately resulting in the fluorescent read-out. Overall, the PDMS-based microfluidic device enabled formation of artificial cells with high throughput, control over vesicle size, biomolecular content, and cell number. In a follow-up study, Trantidou et al. [67] displayed the potential applicability of these artificial cells as biosensors by incorporating *E. coli* genetically equipped with a GFP-coupled *lldPRD* promoter into GUVs to monitor lactate in the external environment of the artificial cell, with a linear measurement range up to 5 mM, in real-time. To circumvent problems associated with the longevity and stability of GUVs, Weiss et al. [68] developed a microfluidic device for the generation of droplet-stabilized GUVs (see Figure 5A). This PDMS-based device enabled sequential loading of transmembrane and cytoskeletal proteins via pico-injection technology as well as subsequent removal of the droplet shell, releasing functional self-supporting protocells into an aqueous, thus physiologically, relevant phase. Exposed to various substrates, protocells that were functionally equipped with integrins displayed distinct differences in their spreading behaviors, thus validating the proteins’ biological functionalities. Upon integration of ATP synthase into the droplet-stabilized GUVs and subsequent exposure to an acidic environment, a total amount of 5 nM ATP could be measured within the released aqueous content of the vesicles. This indicated a functional reconstitution of the enzyme within the stabilized GUVs as well as a correct orientation of at least some of the enzymes within the membrane. Overall, the microfluidic palette was expanded with a powerful tool for the bottom-up assembly of complex synthetic cells, successfully addressing several individual hallmarks simultaneously. In a recent publication, Deshpande et al. [69] (see Figure 5B) presented a novel microfluidic device capable of controllably dividing liposomes with high symmetry and low leakage. Within this device, cell-sized liposomes were generated via octanol-assisted liposome assembly and subsequently flowed against a wedge-shaped splitter, resulting in two liposomes with a size of 6 µm. Octanol-assisted liposome assembly has been previously shown to enable fast maturation times of a few minutes. It also has excellent encapsulation efficiency coupled with the high-throughput production of biologically relevant liposomes in the size range of 5–20 µm. [70] Despite the limitation that this device may not be suitable for multicomponent vesicles, it nonetheless may provide a powerful tool for addressing growth and division cycles of artificial cells. Since not only generation of synthetic cell-like vesicle models (also handling thereof) is a critical aspect in synthetic biology, Yandrapalli et al. [71] integrated a series of micro-structured posts to create a sophisticated PDMS-based device capable of handling up to 23,000 GUVs at once (see Figure 5C). While adjusting the height of the device enables trapping of differently sized subpopulations, it further tunes the assembly of GUVs within different layers in 3D, enabling artificial cell-to-cell interaction studies based on ligand-binding interactions. In addition, this design allows for a precise and fast solution exchange. With only 2 µL, the complete solution around the vesicles can be exchanged, rendering this design a useful tool when working with samples such as nanoparticles, drugs, or proteins. Overall, this chip can be applied for high-throughput experiments capable of delivering statistically robust data sets. Once again, microfluidics is a powerful tool in bottom-up synthetic biology and in the creation of artificial cell-like constructs; however, the question remains whether living cells encapsulated within artificial shells are truly artificial cells made by bottom-up approaches.

## 5. Conclusions and Outlook

**Figure 6:**
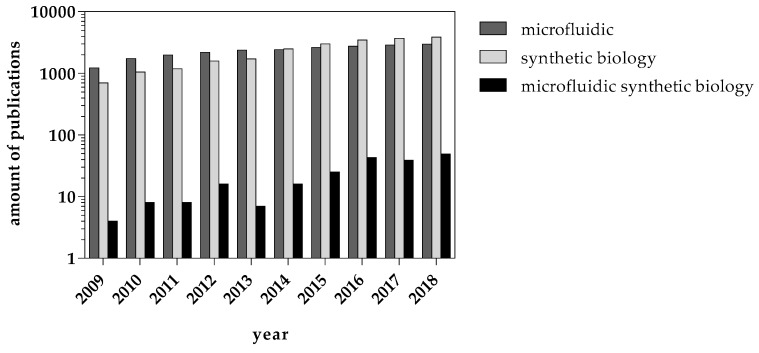
Publication outputs of synthetic biology, microfluidics, and microfluidic synthetic biology over the last ten years expressed as number of total publications (thorough PubMed research using keywords “microfluidic”, ”synthetic biology”, and “microfluidic synthetic biology”).

Commercial gene synthesis and gene construction approaches have become a highly competitive field, where customer demands, including fulfillment time and accuracy, have steadily driven continuous technology improvement. Presently and going forward, there will be tighter correlation and inter-dependency between scale and cost of DNA construction with the need for cycles of iteration to accelerate the growing understanding of underlying complexity and genetic design parameters [72]. One important question to address in synthetic biology is how to increase the predictability of designed artificial systems as novel gene circuits and enzyme libraries. Answering this question will have wide-reaching consequences for the field but will require a shift in how synthetic biology is carried out in academia. Given developments leave no doubt that microfluidics will increase the scope for complexity in the field of bottom-up synthetic biology; however, it has to be noted that up to now, the generation of an artificial cell satisfying all the hallmarks of life is far from being realized. However, as shown in Figure 6, microfluidic and synthetic biology-driven publications have been continuously increasing in recent years with thousands of papers and reviews in contrast to microfluidic synthetic biology publications. Synthetic biology-on-a-chip is a very small community, yet it has been constantly growing over the last 10 years. Publication output in the last few years has reached a plateau phase since 2016, indicating that aside from droplet generators that have obviously become state-of-the-art to create natural and synthetic vesicles on micro- and nanoscales, the combination of synthetic biology and chips is hard work because of the required tight control over experimental procedures. Hopefully, 2020 is a better year, with more microfluidic devices used not only as droplet-machines but also as valuable tools for cell-based synthetic biology and the creation of artificial cells. Research in this field may one day give answers to long asked questions of fundamental cell biology and life itself.
